# Inclusion Rating by Statistics of Extreme Values and Its Application to Fatigue Strength Prediction and Quality Control of Materials

**DOI:** 10.6028/jres.099.032

**Published:** 1994

**Authors:** Y. Murakami

**Affiliations:** Dept. of Mechanical Science and Engineering, Faculty of Engineering Kyushu University, 6-10-1 Hakozaki, Higashi-ku, Fukuoka, 812 Japan

**Keywords:** fatigue, high strength steel, nonmetallic inclusion, statistics of extreme values

## Abstract

The inclusion rating method by statistics of extreme values (IRMSE) using $\sqrt{area}$ of inclusions as the size parameter enables one to discriminate between current super-clean steels. Moreover, IRMSE enables one to predict the size $\left({\sqrt{area}}_{\max}\right)$ of maximum inclusions contained in domains larger than the inspection domain. The statistical distribution of ${\sqrt{area}}_{\max}$ can be used for the quality control of materials and for the prediction of a scatter band of fatigue strength. Practical procedures of inclusion rating and prediction of a scatter band of fatigue strength are shown.

## 1. Introduction

With the increase in cleanliness of steels, conventional inclusion rating methods are no longer as useful as before, because conventional inclusion rating methods cannot determine the cleanliness of new clean steels. Although the cleanliness of steels has been markedly improved in the last two decades, the fatigue strength of recent clean high strength steels cannot attain the ideal value expected from their high static strength. Nonmetallic inclusions are predominantly the cause of lower fatigue strength even for such clean high strength steels. Thus, in order to predict the fatigue strength behavior and to evaluate quality, we need a new inclusion rating method relevant to recent super-clean steels. The inclusion rating method based on statistics of extreme [1] is most relevant for this purpose. In the following, we call this method Inclusion Rating Method by Statistics of Extreme (IRMSE).

In this study, we shall first show that if we choose an appropriate size parameter for inclusions, the size of inclusions obey the statistics of extreme value theory. The appropriate size parameter is the square root of projected area of the maximum inclusion contained in a standard inspection area or volume, ${\sqrt{area}}_{\max}$. Second, we predict the size of the maximum inclusion which may be contained in a larger area or volume than the standard inspection area and, lastly, we use the size parameter, ${\sqrt{area}}_{\max}$, to predict the scatter band of fatigue strength of hard steels.

The merits of IRMSE, in comparison with conventional methods, are (1) to distinctly discriminate the cleanliness of recent super-clean steels, and (2) to predict the size of larger inclusions contained in a domain larger than the inspection domain. This method is useful for quality control of materials and for improvement of the steel making processes. It also enables one to predict the scatter of the fatigue strength of a large number of mass production products.

## 2. Nonmetallic Inclusions as a Fatigue Fracture Origin

**Fig. 1:**
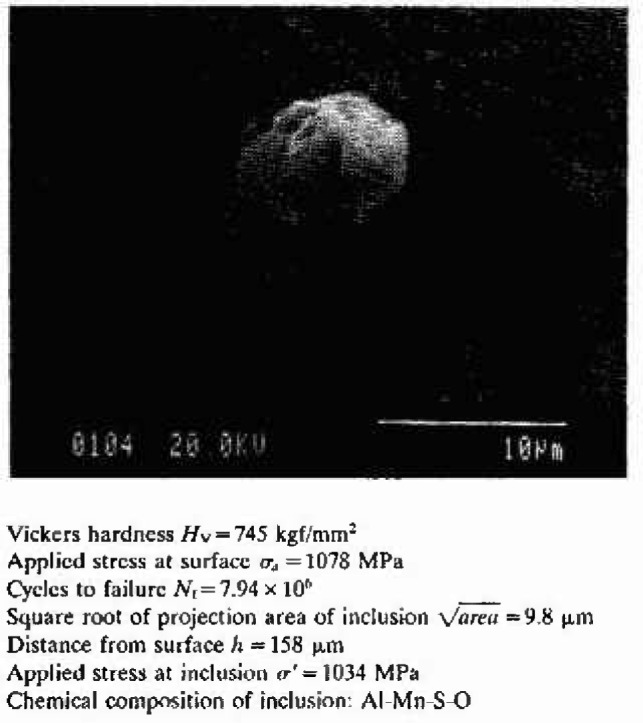
A typical example of inclusion observed at the center of fatigue fracture origin [super-clean bearing steel, SUJ2(N)].

**Fig. 2:**
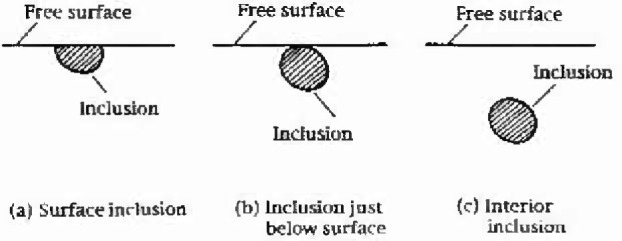
Various locations of inclusions causing fatigue fracture.

Figure 1 shows an example of the nonmetallic inclusion which was observed at fatigue origin of a bearing steel under a rotating bending fatigue test. If this inclusion did not exist in this specimen, the fatigue strength of this specimen should have been higher than the applied stress, *σ_a_* = 1078 MPa. Since the size and location of nonmetallic inclusions scatter randomly, the fatigue strength of high strength steels naturally scatters. Although there has been a firm opinion that the chemical composition and shape of nonmetallic inclusions substantially influences the fatigue limit, Murakami et al. [2–5] have shown the incorrectness of the conventional opinion by their detailed experiments and analyses, and reported distinct experimental evidence that the size of inclusions (defined by $\sqrt{area}$) is the most crucial geometrical parameter. It is empirically known that the intrinsic fatigue strength of steels is determined by the hardness (*H*_v_) of its microstructure. For steels with *H*_v_<400, nonmetallic inclusions contained in current commercial steels are not detrimental and we have the following empirical formula

$$\sigma_{w}≅1.6Hv$$ (1)

where *σ_w_* is the fatigue limit (MPa) and *H*_v_ is the Vickers hardness (kgf/mm^2^). However, for steels with *H*_V_>400, the effect of inclusions reveals itself and the intrinsic or ideal fatigue limit given by Eq. (1) cannot be attained. The fatigue strength depends on the size $\left(\sqrt{area}\right)$ and location of the fatal inclusion and *H*_v_ of the matrix. Murakami et al.’s [6–9] fatigue limit prediction equations are classified into three categories depending on the location of fatal inclusions (see Fig. 2):

Fatigue limit for a surface inclusion [Fig. 2(a)]

$$\sigma_{w}=1.43\;\left(Hv+120\right)/{\left(\sqrt{area}\right)}^{1/6}$$ (2)

Fatigue limit for an inclusion in touch with free surface [Fig. 2(b)]

$$\sigma_{w}=1.41\;\left(Hv+120\right)/{\left(\sqrt{area}\right)}^{1/6},$$ (3)

Fatigue limit for an internal inclusion [Fig. 2(c)]

$$\sigma_{w}=1.56\;\left(Hv+120\right)/{\left(\sqrt{area}\right)}^{1/6},$$ (4)

where the units are *σ_w_*: MPa, $\sqrt{area}$: μ*m*, and *H*_v_: kgf/mm^2^.

Since for a constant value of area, an inclusion is most detrimental when it exists just in touch with the free surface of a specimen, we can use Eq. (3) in combination with the maximum size ${\sqrt{area}}_{\max}$ obtained by IRMSE to predict the lower bound (*σ_wl_*) of scattered fatigue strength of many specimens or machine elements.

## 3. Inclusion Rating of Various High Strength Steels by Statistics of Extreme

**Fig. 3:**
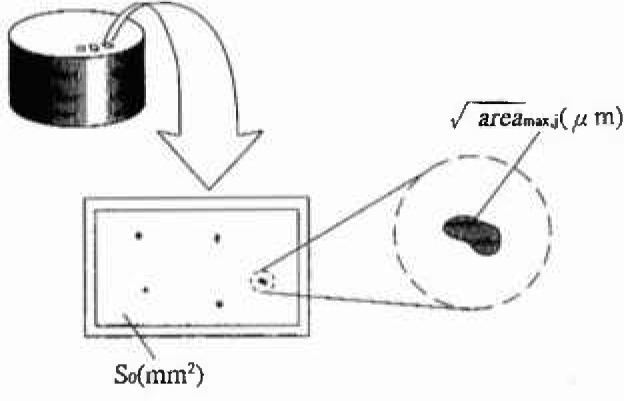
Practical procedure of the inclusion rating by statistics of extreme values.

Figure 3 explains the practical procedure to implement the inclusion rating by statistics of extreme values. The details of this method are reported in Murakami et al.’s papers [3, 5, 9–11]. The procedure is briefly explained in the following, see [11].

**Fig. 4:**
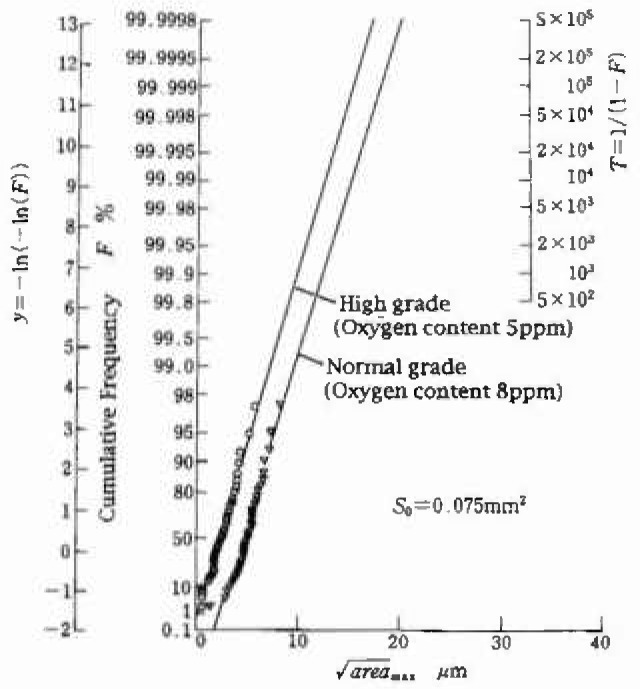
Cumulative frequency of the extreme values of inclusions [Super-clean bearing steels, SUJ2(N) and SUJ2(H)].

1. A section perpendicular to the maximum principal stress is cut from the specimen. After polishing with a n°2000 emery paper, the test surface is mirror-finished with buff.
2. A standard inspection area *S*_0_ (mm^2^) is fixed. Generally, it is advisable to take a microscope picture for an area approximately equivalent to *S*_0_. In the area *S*_0_, the inclusion of maximum size is selected. Then, the square root of the projected area ${\sqrt{area}}_{\max}$ of this selected inclusion is calculated. This operation is repeated *n* times (in *n* areas *S*_0_) (see Fig. 2).
3. The values of ${\sqrt{area}}_{\max j}$ are classified, starting from the smallest, and indexed: (with *j*= 1…*n*). We then have the following relation:
  $${\sqrt{area}}_{\max,1}⩽{\sqrt{area}}_{\max,2}⩽\ldots ⩽{\sqrt{area}}_{\max,n}.$$
  The cumulative distribution function *F_j_* and the reduced variates *y_j_* are then calculated from the equations.
  $$\begin{array}{c} F_{j}=j\times 100/\left(n+1\right)\\ y_{j}=-\ln\left[-\ln(j/\left(n+1\right)\right]. \end{array}$$
4. The data are then plotted on probability paper. The point *j* has an abscissa coordinate of ${\sqrt{area}}_{\max j}$ while the ordinate axis represents either *F_j_* or *y_j_* An example of the curve is shown in Fig. 4.

Figure 4 shows the inclusion ratings by IRMSE for two kinds of super-clean bearing steels, SUJ2(N) and SUJ2 (H). The total oxygen contained in these steels is 8 ppm for SUJ2(N) and 5 ppm for SUJ2(H). This kind of information enables one to discriminate quantitatively the difference among the cleanliness levels of the same kind of materials produced by different companies or produced by a company at different periods. Thus, this information will be useful for the quality control of materials and the improvement of the steel making process.

**Fig. 5:**
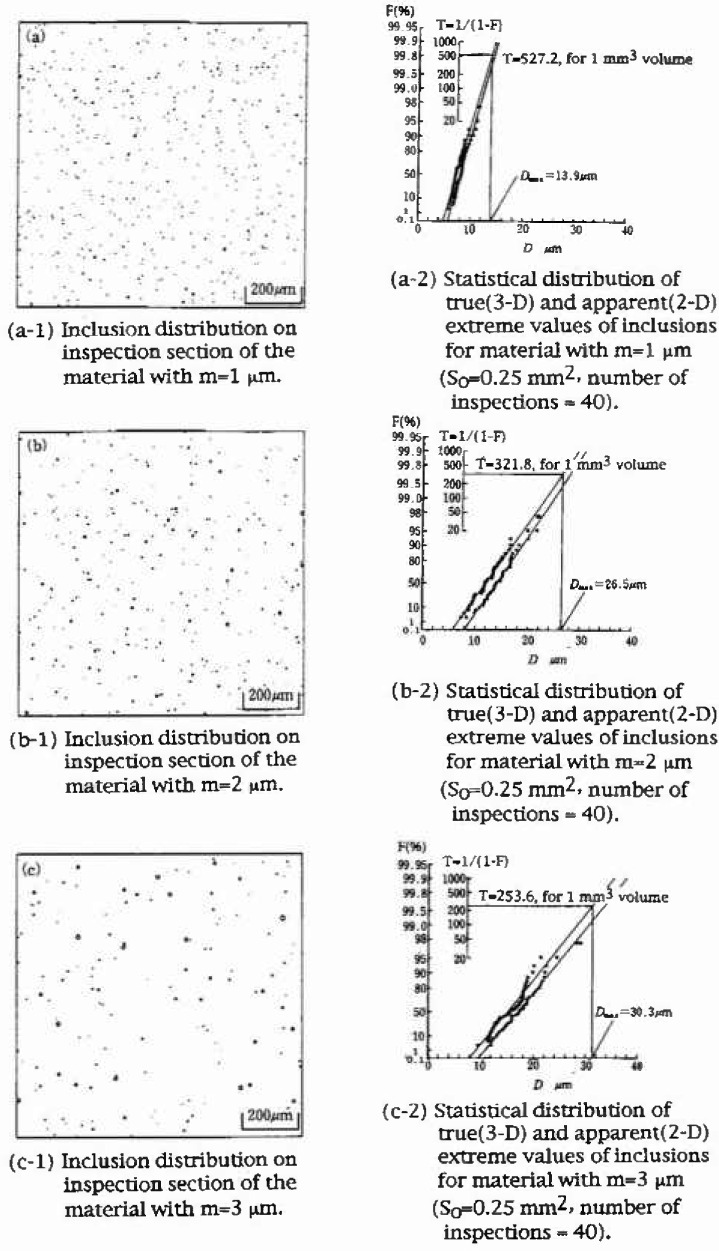
Numerical simulation of the inclusion rating by statistics of extreme values on the materials with the inclusion size distribution of the type $\phi \left(D\right)=\frac{1}{m}\;\exp\;\left(-\frac{D}{m}\right)$.

It is not *a priori* evident to what extent the extreme values ${\sqrt{area}}_{\max}$ of inclusions contained in various steels follow extreme statistics value. However, Murakami et al. [3, 5–11] have shown many examples of measurements which obey the statistics of extreme value theory. Uemura and Murakami [12] carried out a three-dimensional numerical simulation to find the statistical distribution of the extreme values ${\sqrt{area}}_{\max}$ of inclusions which were distributed in a constant volume with the size (*D*) distribution of the type,

$$\phi \left(D\right)=\frac{1}{m}\;\exp\;\left(-\frac{D}{m}\right),$$

where *m* is the mean value, and they confirmed the validity of IRMSE (Fig. 5). In addition, they indicated the quantitative difference between two-dimensional and three-dimensional measurements, though the difference virtually vanishes with increasing inspection domains.

## 4. Application to Prediction of Scatter Band of Fatigue Strength

**Fig. 6:**
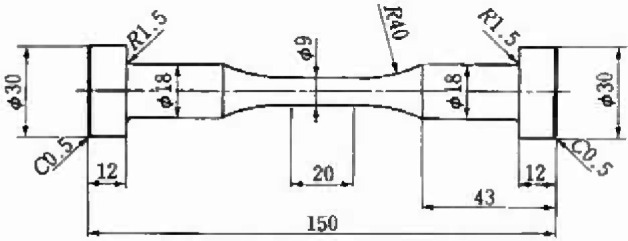
Shape and dimension of tension-compression fatigue specimen (mm) (Tool steel, SKH51).

Figure 6 illustrates the shape and dimension of a tension-compression fatigue specimen [13]. The material used is tool steel, SKH51. The chemical composition is shown in Table 1. Table 2 shows the mechanical properties.

**Fig. 7:**
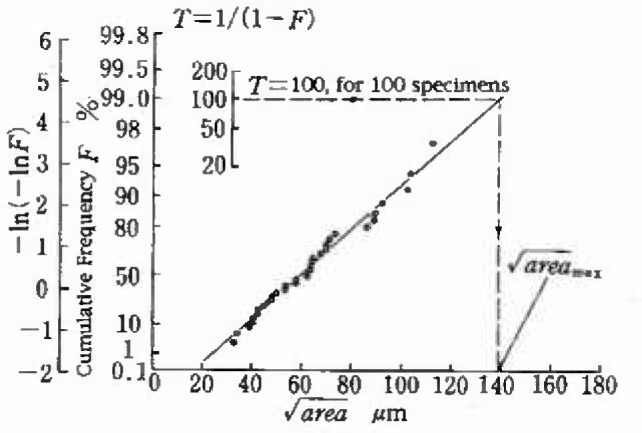
Statistical distribution of the extreme values, the maximum size of inclusion at the center of fracture origin (Tool steel, SKH51).

**Fig. 8:**
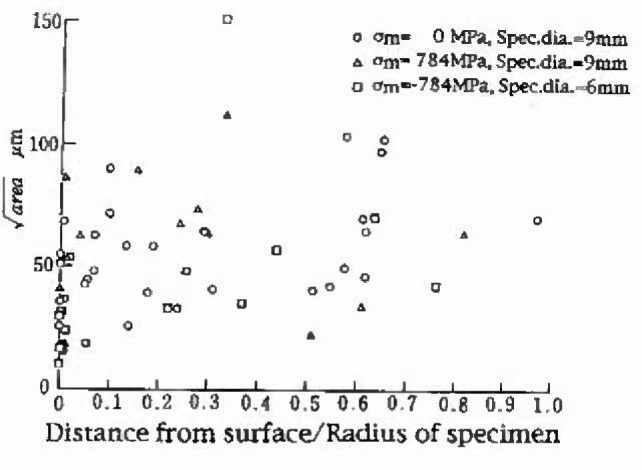
Relationship between the size $\left(\sqrt{area}\right)$ and location of inclusions at the center of fracture origin (Tool steel, SKH51).

Figure 7 shows the extreme value distribution of $\sqrt{area}$ of the inclusions found at the fracture origin of 34 specimens. The data in Fig. 7 are the extreme values obtained by the fatigue test but not by the two-dimensional metallographic method described in Sec. 3. Figure 8 indicates the location of these inclusions on the fracture surface. If the tension-compression fatigue test is not performed correctly, that is, specimens are subject to a bending moment due to a bad alignment or the curving of the specimen axis, nonmetallic inclusions existing near the free surface are likely to appear as the fracture origin on the fracture surface [14]. In such a case, unusually low fatigue strength is likely to be obtained. Since the fatigue fracture origins shown in Fig. 8 are distributed randomly on the section of specimen, these data may be valid for the statistical analysis. However, it should be noted that when the surface inclusions became the fracture origins, the data were not plotted on Fig. 7, because such inclusions are more detrimental than an inclusion having the same size and existing internally and accordingly they may be a little smaller than the exact maximum inclusion.

In the case of the data of Fig. 7, the volume of the test part of one specimen (Fig. 6) corresponds to one inspection domain and there are 34 extreme values in Fig. 7. Therefore, Fig. 7 can be used for predicting the expected maximum size of the inclusion which may be contained in more specimens than those used in fatigue tests. For example, an inclusion having ${\sqrt{area}}_{\max}≅138$ μm is expected to be contained in 100 specimens (N = 100). Combining this ${\sqrt{area}}_{\max}\left(=138\;\mu m\right)$ and Eq. (3), the lower bound (*σ_wl_*) of fatigue strength of 100 specimens can be predicted.

**Fig. 9:**
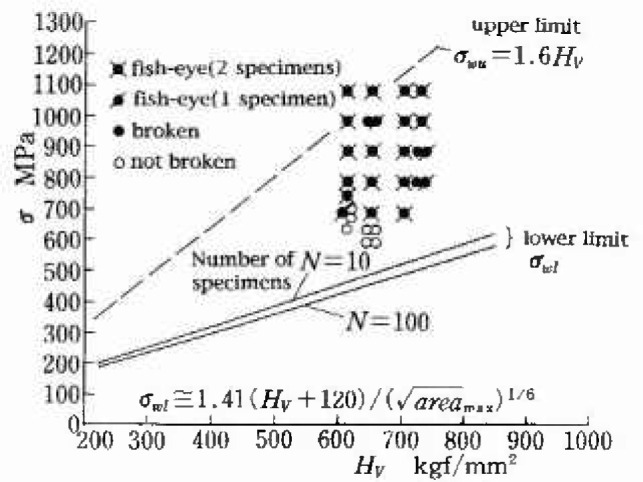
Comparison between the experimental results and the lower bound of fatigue strength which was predicted on the basis of Eq. (3) and the maximum size of inclusion (Tool steel, SKH51).

Figure 9 compares the scatter observed in experiments and the predicted lower bound *σ_wl_* of the scatter band. The prediction is in good agreement with experiments. The prediction of the lower bound of fatigue strength explained above can be used for the quality control of machine elements which are produced by mass-production and cannot be tested individually.

The data as shown in Fig. 7 offer us reliable information on inclusions expected to be contained in other specimens. However, obtaining the data shown in Fig. 7 requires preparation of many precise specimens and time consuming fatigue tests. To avoid this inconvenience, the author has proposed an alternative two-dimensional method as explained in Sec. 3. A sufficient number (TV) of inspection domains (inspection areas) necessary to predict reliably ${\sqrt{area}}_{\max}$ for more specimens or larger areas should depend on the materials to be inspected and on the inspection area *S*_0_ observed by the image processor combined with an optical microscope. From the author’s experience, it is recommended that *N* be larger than 40 for *S*_0_ = 0.031 mm^2^.

Several Japanese industries have already put the method proposed in this study in practice [15].

## 5. Conclusions

1. If we define the size of nonmetallic inclusions contained in commercial steels by the square root of the projected area, $\sqrt{area}$ the maximum values, ${\sqrt{area}}_{\max}$ in a definite inspection domain obey the statistics of extreme value theory.
2. The inclusion rating method by the statistics of extreme values (IRMSE) based on ${\sqrt{area}}_{\max}$ can be used for a new inclusion rating method. IRMSE enables one to discriminate distinctly between recent super-clean steels, while conventional inclusion rating methods are no longer valid as the method to evaluate the cleanliness of new clean steels.
3. IRMSE is useful not only for a relative evaluation of materials but also for the prediction of the expected maximum size of inclusions to be contained in a domain larger than the inspection domain. The value of ${\sqrt{area}}_{\max}$ can be used with the fatigue strength prediction equation to predict a scatter band of fatigue strength of high strength steels.

## Figures and Tables

**Table 1:** Chemical composition in wt% of material (Tool Steel, SKH51)

C	Si	Mn	P	S	Cr	W	Mo
0.81	0.31	0.29	0.018	0.002	3.92	6.10	4.85

V	Co	Cu	Ca	Al	Mg	O	

1.81	0.46	0.07	0.004	0.035	0.0005	0.0018	

**Table 2:** Mechanical properties of quenched and tempered test material (Toot steel, SKH51)

Heat treatment	0.2% Proof stress (MPa)	Tensile strength (MPa)	Elongation (%)	Reduction of area (%)	Vickers hardness *H_v_* (kgf/mm^2^)
Heat treat. 1	1820	2110	2.9	3.7	615
Heat treat. 3	2270	2560	2.0	0	654
